# The effects of somatostatin analogues on liver volume and quality of life in polycystic liver disease: a meta-analysis of randomized controlled trials

**DOI:** 10.1038/s41598-021-02812-z

**Published:** 2021-12-06

**Authors:** Carlo Garofalo, Ivana Capuano, Luigi Pennino, Ilaria De Gregorio, Eleonora Riccio, Michele Provenzano, Felice Crocetto, Pasquale Buonanno, Savio Domenico Pandolfo, Michele Andreucci, Antonio Pisani

**Affiliations:** 1 Division of Nephrology, Department of Scienze Mediche e Chirurgiche Avanzate, University of Campania “Luigi Vanvitelli”, Via M. Longo 50, 80138 Naples, Italy; 2 Department of Public Health, Chair of Nephrology, University Federico II of Naples, Naples, Italy; 3 Institute for Biomedical Research and Innovation, National Research Council of Italy, Palermo, Italy; 4 Division of Nephrology at Department of Health Sciences, “Magna Graecia” University, Catanzaro, Italy; 5 Department of Neurosciences, Reproductive and Odontostomatological Sciences, University of Naples Federico II, Naples, Italy

**Keywords:** Gastroenterology, Nephrology

## Abstract

A clear evidence on the benefits of somatostatin analogues (SA) on liver outcome in patients affected by polycystic liver disease is still lacking. We performed a meta-analysis of RCTs and a trial sequential analysis (TSA) evaluating the effects of SA in adult patients with polycystic liver disease on change in liver volume. As secondary outcome, we evaluated the effects on quality of life as measured by SF36-questionnaire. Six RCTs were selected with an overall sample size of 332 adult patients with polycystic liver disease (mean age: 46 years). Mean liver volume at baseline was 3289 ml in SA group and 3089 ml in placebo group. Overall, unstandardized mean difference in liver volume was − 176 ml (95%CI, − 406, 54; *p* < 0.133). Heterogeneity was low (I^2^:0%, *p* < 0.992). However, we performed a moderator analysis and we found that a higher eGFR significantly correlates to a more pronounced effect of SA on liver volume reduction (*p* = 0.036). Cumulative Z-curve in TSA did not reach either significance and futility boundaries or required information size. Three RCTs have evaluated Quality of life parameters measured by SF36-QOL questionnaire for a total of 124 patients; no significant difference was found on the effect of SA on QOL parameters when compared with placebo. The present meta-analysis revealed a potential effect of SA on reduction of liver volume and quality of life parameters, but results did not reach a statistical significance. These data could be explained by the need of further studies, as demonstrated through TSA, to reach an adequate sample size to confirm the beneficial outcomes of SAs treatment.

## Introduction

Autosomal dominant polycystic kidney disease (ADPKD) is the most frequent hereditary kidney disease, generally caused by the mutation of PKD1 and PKD2 genes, and more rarely by other two genes as GANAB^1^ and DNAJB11, recently identified^2^. ADPKD is characterized by the development and growth of cysts in the kidney with onset of chronic kidney disease (CKD) and it accounts for 8–10% of end-stage kidney disease (ESKD) cases; the disease is also characterized for systemic manifestations with other organs involvement^3–5^.

The polycystic liver disease (PLD), defined by the presence of more than 10 liver cysts^6^, is a common extrarenal manifestation in ADPKD with a prevalence of about 80%, that increases to more than 90% in elderly subjects^7^. PLD can also occur as an isolated entity with limited or absent cysts in the kidneys, the autosomal dominant polycystic liver disease (ADPLD), caused by mutations in the PRKCSH and SEC63 genes (responsible for the half of isolated PLD)^8,9^. Most PLD patients are asymptomatic, but some could develop hepatomegaly associated with abdominal pain, post-prandial early satiety, dyspnea and worsening in quality of life (QoL)^10^.

Until very recently, there were no available disease-modifying therapies, thus limiting ADPKD treatment to the management of its complications. This represents an important issue when considering that ADPKD is associated with high-risk for CKD progression and cardiovascular damage^11,12^. The treatment of liver cysts is limited to invasive interventions such as aspiration, sclerotherapy, or fenestration of cysts up to restricted liver resection; however, these treatments do not limit disease progression^13–15^.

Some treatments targeting cyclic AMP (cAMP) signaling showed to be effective on kidney outcomes; in particular, tolvaptan is the only drug, worldwide available, approved by regulatory agencies to slow renal growth and the decline of kidney function in ADPKD patients (aged 18–55 with stage 1–4 CKD and a rapid progression of the disease); conversely, somatostatin analogues (SA) could slow the growth of the kidneys in ADPKD, but no effect on the glomerular filtration rate decline has been demonstrated. However, in the last few years, several trials have demonstrated the liver volume reducing effects of SA in patients with polycystic liver disease^6, 16–20^. A recent meta-analysis has confirmed the beneficial effect from SAs on liver volume when compared to placebo. However, in this study some results were derived without an access to original data^21^. Furthermore, during the last year another RCT with a newer SA, pasireotide, was published^22^. On this basis, our meta-analysis aimed to investigate the real effects of SAs on liver volume, and it is the first work which explored the effects of SAs on QoL in PLD.

## Materials and methods

### Search strategies

The present meta-analysis was conducted according to the Preferred Reporting Items for Systematic reviews and Meta-Analyses (PRISMA) guidelines^23^. Relevant articles published until 31 March 2021 were searched using two large databases (PubMed and ISI Web of Science) without language restriction. These Medical Subject Headings (MeSH) and text words were used: ADPKD, ADPLD “polycystic liver”, PLD and “somatostatin analogues”, SA, octreotide, lanreotide or pasireotide (item S1). To identify other potentially relevant studies, references of articles and reviews found in our research were screened. This systematic review was registered on PROSPERO (CRD42020223490).

### Study selection

We selected studies on the base of the following inclusion criteria: (1) RCTs evaluating, as primary outcome, the changes on liver volume of SA compared with placebo (or standard care) in patients affected by polycystic liver disease; (2) liver volume evaluated by CT scan or MRI. We also evaluated as secondary outcomes difference in quality of life (QOL) parameters measured by SF36 QOL questionnaire (physical functioning, social functioning, physical role, emotional role, mental health, vitality, bodily pain, change in health perception, general health perception, physical component summary, mental component summary) between patients treated by SA and placebo (or standard care). Finally, adverse effects related to SA were reported.

Reduction in liver volume was calculated as the difference between liver volume from baseline to the end of intervention in SA and placebo/standard care groups. The titles and abstracts of each article found using the same search strategy, were screened independently by two investigators (CG and LP). The full paper of relevant studies was obtained, and each manuscript was reviewed using predefined eligibility criteria. Where the data from primary studies were incomplete or unavailable in required form, the Authors of manuscripts were directly contacted. Any discrepancy between the two authors on study eligibility was resolved through consensus agreement. Data extraction was performed independently by the two authors using predefined forms.

### Assessment of risk of bias in included studies

The risk of bias in the included RCTs was evaluated with the risk of bias assessment tool^24^. Nine items associated with the risk of bias were evaluated: adequate random sequence generation, allocation concealment, blinding of participants, blinding of assessment, incomplete outcome data adequately addressing, selective outcome reporting, other sources of bias, risk of carry over effect, and potential bias from confounding factors^24^.

### Statistical analysis

Agreement for study selection and quality assessment were quantified. To assess the effects of treatment, the unstandardized mean difference (UMD) was used to compare the difference in total liver volume from baseline to end of treatment in patients treated by placebo/standard care or SA with a pre-post correlation of 0.5; analyses were also repeated considering a pre-post correlation of 0.7 and 0.9. When in the studies values were reported as median and interquartile range or mean and 95%CI, Authors were directly contacted to have original data as mean and standard deviation. In cross-over studies, we considered as the mean difference in outcomes the difference between the end of placebo and SA periods. Extracted results were pooled with a conservative approach by using random-effects model. We analyzed heterogeneity with the I^2^ statistic with 95% CI^25^. I^2^ values of 25%, 50%, and 75% correspond to cut-off points for low, moderate, and high degrees of heterogeneity. Sensitivity analyses were conducted to exclude that a study was exerting excessive influence on the heterogeneity^26^. Moderator analyses and univariate random-effects meta-regression were performed to explore other sources of heterogeneity. Moderators evaluated were sample size (< 50 or ≥ 50 subjects), follow-up duration (≤ 1 or > 1 year), prevalence of female gender (< 50% or ≥ 50%), baseline GFR (< 60 or ≥ 60 ml/min), study drug (Octreotide or Lanreotide *versus* Pasireotide), liver volume evaluation (by CT or MRI), baseline liver volume (< 2000 or ≥ 2000 ml), presence of patients with isolated PLD in the cohort (yes vs no). Meta-regression was performed to test influence of GFR and percentage of female gender used as continuous variables. Restricted maximum likelihood estimators were used to estimate model parameters^27^. Begg’s rank correlation test and Egger’s linear regression were used to assess the publication bias^28^. Two-sided *p *value < 0.05 is considered significant. Analyses were performed using PROMETA 2 (INTERNOVI, Cesena, Italy), STATA/SE 11 (Stata Corporation, College Station, TX, USA) and RStudio version 1.1442 (RStudio: Integrated development environment for R. Boston, US). Trial sequential analysis (TSA) was performed using the software TSA version 0.9.5.10 Beta (Copenhagen Trial Unit, Centre for Clinical Intervention Research, www.ctu.dk/tsa); the required information size (RIS) was calculated with α = 0.05, β = 0.20.

## Results

**Figure 1:**
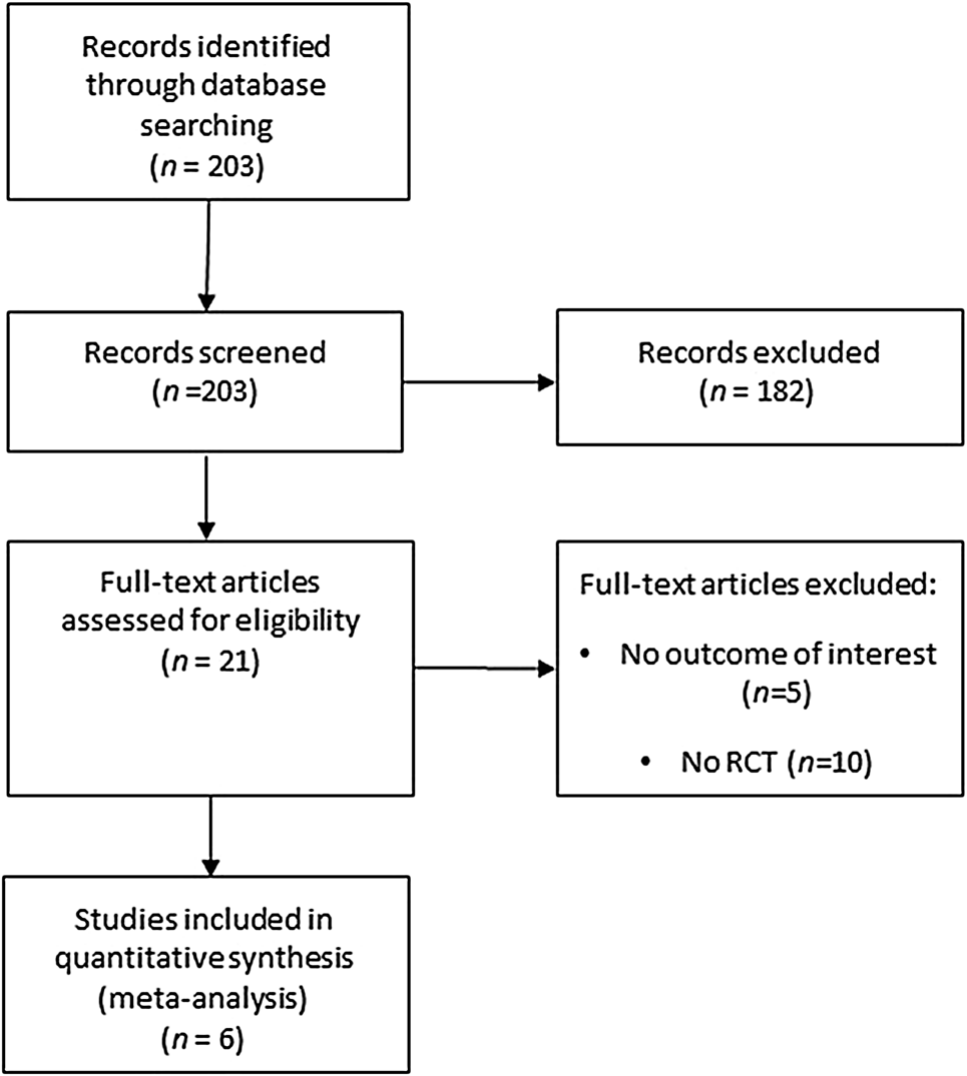
Flow-chart of study selection.

After the screening of titles and abstracts carried out from search strategy, 21 studies out of 203 were considered potentially relevant. The full text of each of these articles was reviewed by two authors, and 6 eligible studies^6,16–20,22^ were included in the meta-analysis (Fig. 1). The agreement for study selection was very good as testified by Kappa value of 0.934.

### Patient characteristics

**Figure 2:**
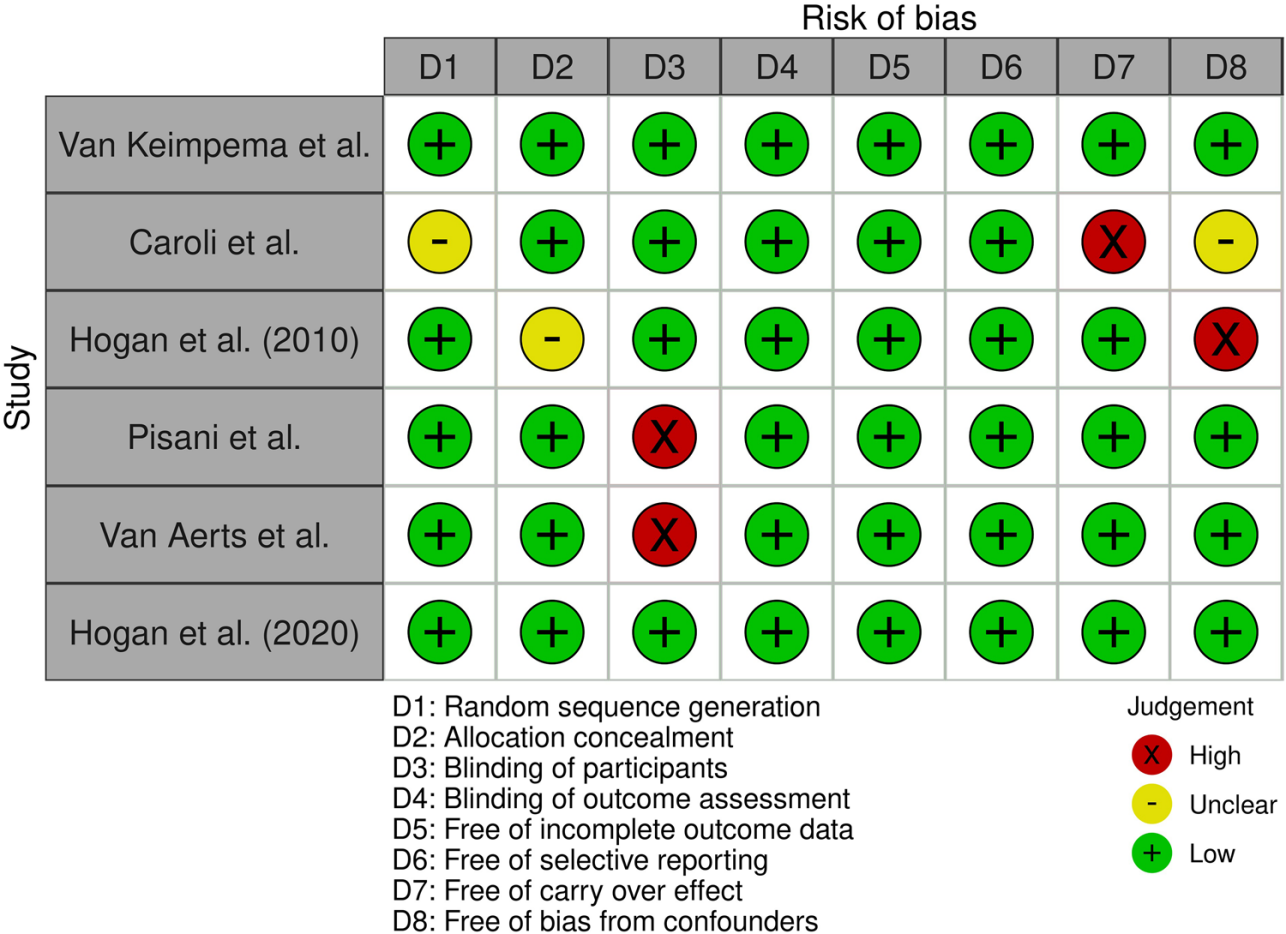
Risk of bias in RCTs included in meta-analysis.

Selected RCTs are summarized in Table 1. Overall, studies included information on 332 individuals. The sample size of these studies ranged from 12 to 157 participants. No study included Asian populations while all included Western populations (2 studies from Italy, 2 from Netherlands and 2 from US). In three studies the SA used was octreotide, in two lanreotide and in one pasireotide. Study duration was ≥ 52 weeks in four studies. Mean age was 46.0 years while mean eGFR was 68.6 ml/min/1,73 m^2^. The risk of bias was low in all included studies (Fig. 2).

**Table 1:** Demographic and clinical characteristics of cohorts included in meta-analysis.

Authors	Country (Year)	Nr. Patients SA/Placebo	Study design	Intervention duration (weeks)	Mean age (years)	Female gender (%)	Mean eGFR (ml/min)	Study drug	Liver volume scan	Presence of ADPLD	Outcomes
Van Keimpema et al	Netherlands (2009)	27/27	Randomized, double-blind, placebo-controlled trial	24	49.6	87	69 (MDRD)	Lanreotide 120 mg subcutaneously every 28 days	CT	Yes	TLV, HRQL
Caroli et al	Italy (2010)	12/12	Post-hoc analysis of a randomized, crossover, placebo-controlled trial	24	44.5	25	57 (iohexol clearance)	Octreotide LAR 40 mg intramuscularly every 28 days	CT	No	TLV
Hogan et al	US (2010)	28/14	Randomized, double-blind, placebo-controlled trial	52	49.7	86	70 (iothalamate clearance)	Octreotide LAR 40 mg intramuscularly every 28 days	MRI/CT	Yes	TLV, HRQL
Pisani et al	Italy (2016)	14/13	Post-hoc analysis of a randomized controlled trial	156	33.4	63	92 (iohexol clearance)	Octreotide LAR 40 mg intramuscularly every 28 days	MRI	No	TLV
Van Aerts et al	Netherlands (2019)	83/74	Secondary analysis of a randomized controlled trial	120	48.2	53	51 (MDRD)	Lanreotide 120 mg subcutaneously every 28 days	MRI	No	TLV
Hogan et al	US (2020)	29/12	Randomized, double-blind, placebo-controlled trial	52	50.8	93	72 (CKD-EPI)	Pasireotide LAR 60 mg intramuscularly every 28 days	MRI	Yes	TLV, HRQL

*SA* somatostatin analogue, *LAR* long-acting release, *CT* computed tomography, *MRI* magnetic resonance imaging, *TLV* total liver volume, *TKV* total kidney volume, *eGFR* estimated glomerular filtration rate, *HRQL* health-related quality of life, *ADPLD* autosomal dominant polycystic liver disease.

### Outcomes

#### Liver volume

**Figure 3:**
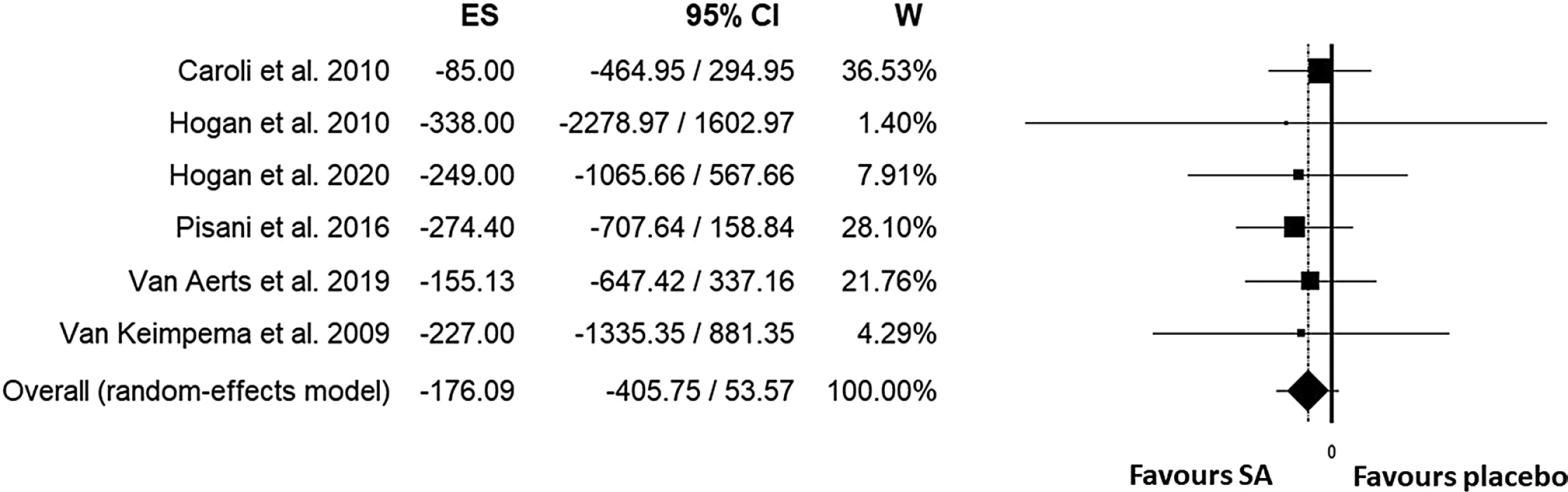
Unstandardized mean difference in liver volume comparing somatostatin analogues and placebo/standard management.

**Figure 4:**
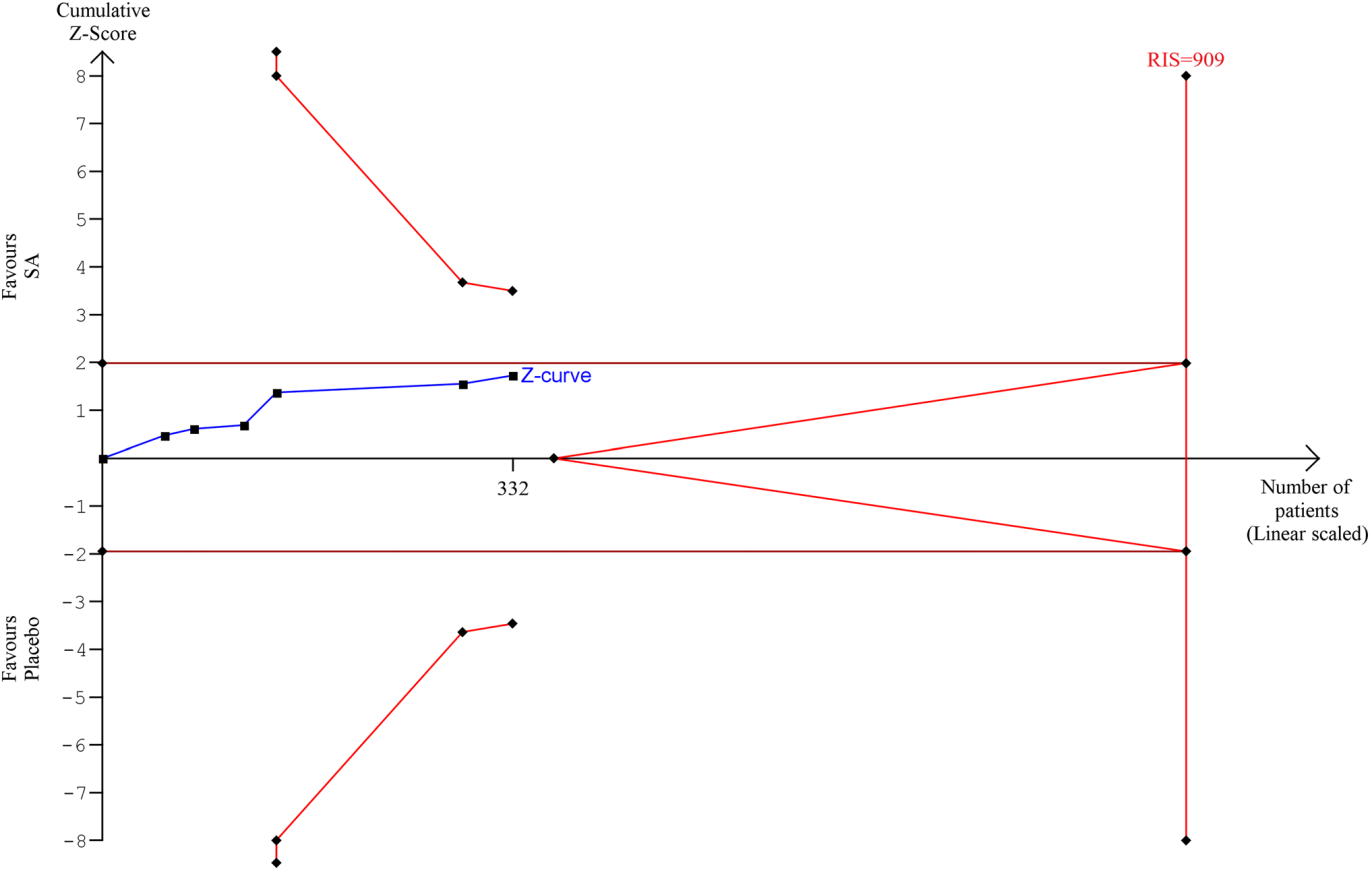
Trial sequential analysis for meta-analysis of effect of somatostatin analogues on liver volume.

All included trials evaluated liver volume in subjects treated with a somatostatin analogue or placebo. In subjects treated with somatostatin analogues (SA) the mean basal liver volume was 3289 ml while in placebo group 3089 ml. Unstandardized overall mean difference in total liver volume comparing SA and placebo/standard care was − 176 ml (95%CI, − 406, 54; *p* < 0.133) (Fig. 3). Heterogeneity was low (I^2^: 0%, *p* < 0.992). In sensitivity analysis, no study exerted a significant influence on the results. When we evaluated moderator analysis (Table 2), we did not find any significant role for prevalence of female gender (≥ or < 50%), number of enrolled subjects (≥ or < 50), study duration (> or ≤ 52 weeks), use of CT or MRI to detect liver volume, baseline liver volume (≥ or < 2000 ml), baseline eGFR (< or ≥ 60 ml/min), study drugs (octreotide/lanreotide *vs* pasireotide) mean age, mean basal liver volume, publication year, presence of patients with isolated PLD in the cohort. In meta-regression analysis, a significant role was found for baseline GFR with a greater reduction in liver volume due to the effects of SA for higher eGFR, *p* = 0.036 (Supplemental Fig. 1) while no significant role was found for female gender percentage (Supplemental Fig. 2). No publication bias was found as testified by Funnel plot (Supplemental Fig. 3), Begg’s test: (*p* = 0.999) and Egger’s test: (*p* = 0.244). Figure 4 shows the TSA for meta-analysis of effect of SAs on liver volume: cumulative Z-curve does not reach either significance and futility boundaries or optimal sample size (RIS = 909).

**Table 2:** Subgroup analyses of somatostatin analogues effects on liver volume as compared to placebo/standard management.

Subgroup	Unstandardized mean difference (95% CI)	*P*
**Sample size**		0.951
< 50	− 171 (− 458, 112)	
≥ 50	− 186 (− 580, 208)	
**Follow-up**		0.694
≤ 1 year	− 130 (− 454, 194)	
> 1 year	− 222 (− 548, 103)	
**Female Gender**		0.555
< 50%	− 85 (− 465, 295)	
≥ 50%	− 229 (− 517, 60)	
**Baseline eGFR**		0.512
< 60 ml/min	− 111 (− 412, 190)	
≥ 60 ml/min	− 267 (− 622, 89)	
**Study drug**		0.855
Octreotide LAR/Lanreotide	− 170 (− 409, 69)	
Pasireotide	− 249 (− 1066, 568)	
**Liver volume evaluation**		0.589
CT	− 99 (− 459, 259)	
MRI	− 228 (− 527, 70)	
**Baseline liver volume**		0.920
< 2000 ml	− 167 (− 453, 118)	
≥ 2000 ml	− 192 (− 578, 194)	
**Presence of patients with isolated PLD**		0.799
Yes	− 251 (− 874, 371)	
No	− 164 (− 411, 83)	

*eGFR* estimated glomerular filtration rate,* CI* confidence interval,* CT* computed tomography, * MRI* magnetic resonance imaging, * PLD* polycystic liver disease.

#### Quality of life

**Figure 5:**
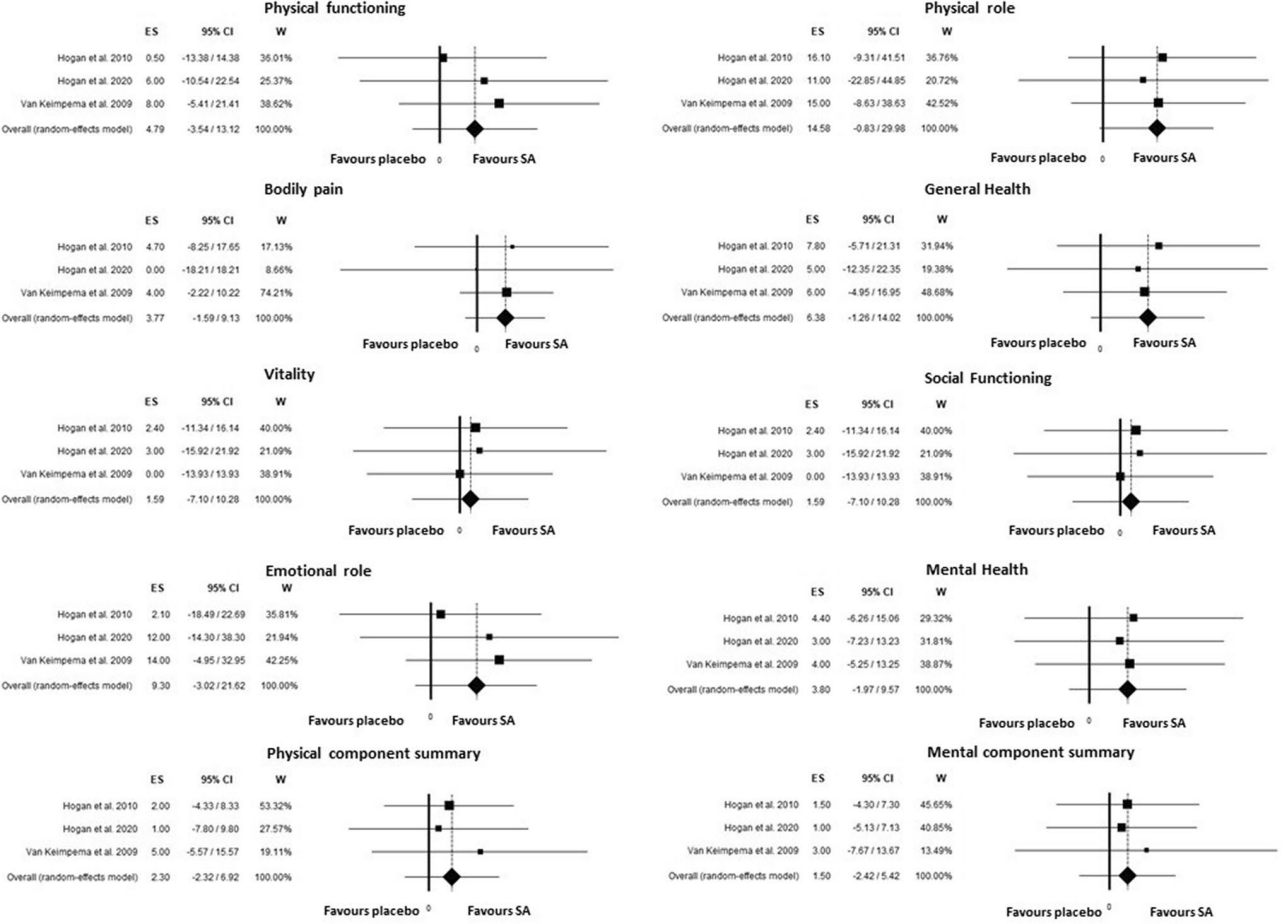
Unstandardized mean difference in quality-of-life parameters measured by SF36-QOL questionnaire comparing somatostatin analogues and placebo/standard management.

Three RCTs have evaluated Quality of life parameters measured by SF36 QOL questionnaire for a total of 124 patients evaluated (74 and 50 treated by SA and placebo/standard care respectively)^17, 18, 22^. As evidenced in Fig. 5, no significant difference was found on the effect of SA on QOL parameters when compared with placebo group. The difference in physical role was the only marginally significant (*P* = 0.06) with slight improvement in SA group (15, 95%CI, − 1–30). No significant heterogeneity was found in each parameter and no publication bias was found.

#### Adverse effects

In the studies included in meta-analysis no severe adverse effects related to the treatment with somatostatin analogues were reported (Table 3). Most common side effects related to SA therapy were gastrointestinal and included diarrhea (27.5%); abdominal cramping/bloating (20.2%), asthenia (10.4%), injection site pain (10.9%). Mean fasting plasma glucose levels increased only in group treated with pasireotide in study by Hogan et al. 2020^22^ with prevalence of hyperglycemia of 23.8% while remained stable in the placebo/standard care group.

**Table 3:** Adverse effects during RCT related to SA vs placebo/standard management.

Type of adverse effects	Number of events in placebo/standard management group	%	Number of events in SA group	%
Gastroenteritis	1	0.66%	0	0.0%
Nephrolithiasis	1	0.66%	0	0.0%
Hypertension	1	0.66%	0	0.0%
Surgical complications	1	0.66%	0	0.0%
Constipation	0	0.00%	1	0.5%
QT > 480 ms	0	0.00%	1	0.5%
Hernia	1	0.66%	1	0.5%
Acute cholecystitis	0	0.00%	1	0.5%
Fever	0	0.00%	1	0.5%
Intracranial aneurysm	0	0.00%	1	0.5%
Rash	0	0.00%	2	1.0%
Hepatic cyst hemorrhage	0	0.00%	2	1.0%
Renal cyst hemorrhage	1	0.66%	3	1.6%
Hypoglycemia	0	0.00%	3	1.6%
Cholelithiasis	0	0.00%	4	2.1%
Headache	3	1.99%	5	2.6%
Urinary tract infection	4	2.65%	6	3.1%
Hepatic cyst infection	0	0.00%	6	3.1%
Nausea	3	1.99%	9	4.7%
Alopecia	0	0.00%	11	5.7%
Steatorrhea	0	0.00%	12	6.2%
Bradycardia	1	0.66%	13	6.7%
Abdominal pain	1	0.66%	14	7.3%
Injection site granulomas	0	0.00%	18	9.3%
Asthenia	4	2.65%	20	10.4%
Injection site pain	3	1.99%	21	10.9%
Abdominal cramping/bloating	3	1.99%	39	20.2%
Hyperglycemia	5	3.31%	46	23.8%
Diarrhea	15	9.93%	53	27.5%
Gastrointestinal disorders (total)	23	15.23%	128	66.3%

## Discussion

We performed the first meta-analysis of RCTs investigating the effectiveness of SA on liver volume, using all original data, and its consequences on the QoL in ADPKD patients. Liver volume represents a prognostic marker disease as it affects clinical symptoms and health related QoL. Our results confirm the previous studies even if it does not reach statistical significance; in fact, we found that the overall mean difference in liver volume, including information on 332 patients, was not significant comparing SA and placebo.

A recent meta-analysis, investigating the effects of SA on TKV, TLV and eGFR decline demonstrated a significant reduction of TLV in 311 ADPKD and PLD patients; on the contrary, SAs did not improve neither eGFR or TKV and they did not affect the progression of ESKD^21^. Compared to Griffiths et al., we included an RCT published in 2020 on the effects of pasireotide long-acting release with TLV as primary outcome. Pasireotide is a pansomatostatin more stable agonist with a broader spectrum of affinity for specific receptors. In particular, Hogan et al. conducted a RCT on 41 PLD and ADPKD (n = 29 pasireotide-LAR, n = 12 placebo) suggesting that SAs have a significant and clinically relevant effect of arresting growth of both liver and kidney cysts^22^. Of note, we analyzed original data as mean and standard deviation; on the contrary, Griffiths et al. analyzed derived data of two studies^18,19^, introducing an important bias. In particular, the use of derived data allowed to the study conducted by van Aerts et.al.^19^ to acquire a weight of 85%, so unbalancing the results in favor of the beneficial effects of SA. Moreover, we performed for the first time a TSA confirming that the RCTs so far conducted are insufficient to draw any definitive conclusion about the effect of SA on liver volume, though the single RCTs appeared to suggest a benefit with SA therapy.

On the other hand, in our sensitivity analysis no study exerted a significant influence on the results; therefore, we did not find any significant role for prevalence of female gender evaluated as categorical variable (presence of female > or < 50% in each study, Table 2) and continuous variable (percentage of women, Supplemental Fig. 2), in contrast to what reported in the past, probably due to larger number of female participants in trials where TLV was reported^29^. Moreover, we did not find any significant role for prevalence of number of enrolled subjects, study duration, use of CT or MRI to detect liver volume, baseline eGFR, mean age, study drugs (octreotide vs newer SA), mean basal liver volume, as already Gevers et al. demonstrated. Instead, we found that higher baseline eGFR was associated with a greater reduction in liver volume due to the effects of SAs. Noteworthy, in the previous meta-analysis, when studies were separated by choice of SAs, lanreotide demonstrated a significant reduction in TLV, not observed in the octreotide subgroup^21^. ADPKD is characterized by cyst growth caused by the mutations of multiple genes encoding for specific proteins which regulate cell proliferation and fluid secretion; the cAMP pathway is the main signal transduction pathway involved in the pathogenesis of the disease^30^. Differently from vasopressin whose receptors have no liver expression, SA interact with somatostatin receptors expressed not only on renal tubular cells, but also on cholangiocytes and hepatocytes, with different pharmacodynamic features based on the type of SA; in fact, pasireotide binds to SSTR1,2,3, and 5 with more affinity and stability compared to octreotide.

To our knowledge, this is the first meta-analysis of the effects of SA on QoL in ADPKD and PLD.

Its evaluation was assessed by SF36 QoL questionnaire in three RCTs^17,18,22^ in a total of 124 patients and no significant difference was found on the effect of SA on QoL parameters when compared with placebo group. Interestingly, the difference in physical role was only marginally significant (*p* = 0.06) with slight improvement in SA group. Our results on QoL are different from the study by Neijenhuis that pooled data from only two RCTs and found a significant effect on physical component score associate with SA^31^. However, we included three studies that are the minimum to perform a meta-analysis; furthermore, we could not exclude an additional negative effect of pasireotide side-effects^22^ on QoL that negates the positive effect observed in aforementioned study.

Van Aerts et al., based on a review of the current literature and their clinical experience, asserted that polycystic liver with a heighted total liver volume lower than 1600 ml is asymptomatic and patients can occasionally experience pain located at the back and flank determined by the stretch of liver capsule^6^. Patients with a heighted total liver volume more than 1600 ml are at higher risk to develop compression symptoms and the disease is classified as moderate. Liver can be palpated below the left costal margin and symptoms come from intestinal, stomach and lung compression with patients experiencing pain, dyspnea, early satiety, and gastro-esophageal reflux. In the severe stage, these symptoms are extremely emphasized with a prominent belly causing psychological problems too. Along with the above-mentioned compression symptoms, which can also affect liver function by the obstruction of hepatic veins, complications could also derive from cysts’ hemorrhage, infection, and rupture. For decades, treatment options relied on cyst aspiration and sclerotherapy, fenestration, resection, and liver transplantation, the latter represents the only definitively curative option, but only a minority of patients is eligible for the intervention. The absence of evidence of efficacy on hepatic outcome and the association of hepatotoxicity of treatment with tolvaptan may limit its use in PLD and make somatostatin analogs the suitable alternative. However, in our meta-analysis no significant difference in liver volume was found when we performed a sensitivity analysis excluding the study by Caroli et al.^16^ that included patients with a baseline total liver volume < 1600 ml.

The most common adverse events of SA treatment are diarrhea, abdominal pain, cholelithiasis, and cholecystitis, because of the expression of SSTRs at this level, and hyperglycemia caused by the inhibition of SSTR expressed on both insulin producing β-cells and the glucagon-producing α-cells; in the studies included in our meta-analysis no severe adverse effects related to the treatment with SA were reported suggesting a good safety profile (Table 3).

Our study had some limitations. First, we could not exclude that some patients were enrolled in both RCTs by Hogan et al.^17,22^. Second, in study by Hogan et al. 2020^22^ only heighted TLV was available in the manuscript and, for this, reason, we used heighted TLV spite TLV. However, because we evaluated the difference in liver volume from baseline to the end of treatment between placebo (or standard care) and SA, use of difference in heighted TLV from Hogan et al. 2020 RCT could not represent a real confounder. Third, we found that baseline eGFR could influence the relationship between use of SA and difference in liver volume. We could not exclude that this was caused by inclusion of isolated PLD patients that, usually, have normal eGFR given the absence of renal cysts, and might have lower TLV progression compared to ADPKD patients^32^. However, as showed in Table 2 no difference in liver outcome was found comparing studies including both ADPKD and isolated PLD or ADPKD alone. Fourth, the results of our meta-analysis were not significant for total liver volume difference although individual RCTs have suggested a beneficial effect. This difference from original manuscripts was related to the small sample size of each study included, with high standard deviation and consequently high variance values of unstandardized mean difference which makes the results not significant in meta-analysis. Furthermore, when we repeated all analysis considering only final liver volume the meta-analysis was also not significant (data not shown); finally, also considering a pre-post correlation of 0.7 and 0.9 results did not change (Supplemental Table 1).

## Conclusion

SA are the only therapy so far available for the treatment of PLD, but only few RCTs have been conducted to clarify their effect on liver volume. Our meta-analysis showed that the patients treated with SAs experienced a greater reduction in TLV compared to placebo, but this difference was not statistically significant; this result could be explained by the need of further studies, as we demonstrated through TSA, to reach an adequate sample size to draw any definitive conclusion.

Therefore, SA showed potential benefit in the management of PLD, but additional trials will be fundamental to confirm the encouraging trend we observed.

## Supplementary Information

Supplementary Information.

## Data Availability

Data from the present meta-analysis are available on request to the corresponding author.
