# Supplementary material for: The effects of somatostatin analogues on liver volume and quality of life in polycystic liver disease: a meta-analysis of randomized controlled trials
Source: Sci Rep. 2021 Dec 6;11:23500. doi: 10.1038/s41598-021-02812-z (PMC8648823; doi:10.1038/s41598-021-02812-z)
Supplement: Supplementary file 1 — Supplementary Information. [file 41598_2021_2812_MOESM1_ESM.docx]

SUPPLEMENTAL MATERIAL

**Item S1.** Literature search strategy for PubMed and ISI Web of Science

Supplemental Figure 1. Meta regression of differences in glomerular filtration rate and overall unstandardized mean difference of six studies evaluating liver volume.

Supplemental Figure 2. Meta regression of differences in female gender percentage and overall unstandardized mean difference of six studies evaluating liver volume.

Supplemental Figure 3. Funnel plot for publication bias among studies evaluating the effects of somatostatin analogues on liver volume.

Supplemental Table 1. Baseline and final liver volume in somatostatin analogue group and placebo/standard management with values of unstandardized mean difference considering a pre-post correlation of 0.5, 0.7 and 0.9.

**Item S1. Literature search strategy for PubMed and ISI Web of Science**

#1 (("polycystic liver"[Text Word]) OR ("ADPLD"[Text Word]) OR ("PCLD"[Text Word]) OR ("ADPKD"[Text Word])

#2 (("somatostatin analogues"[Text Word]) OR ("SA"[Text Word]) OR ("lanreotide"[Text Word]) OR ("octreotide"[Text Word]) OR ("pasireotide"[Text Word])

#3 (#1 AND #2)

Supplemental Figure 1. Meta regression of differences in glomerular filtration rate and overall unstandardized mean difference of six studies evaluating liver volume.


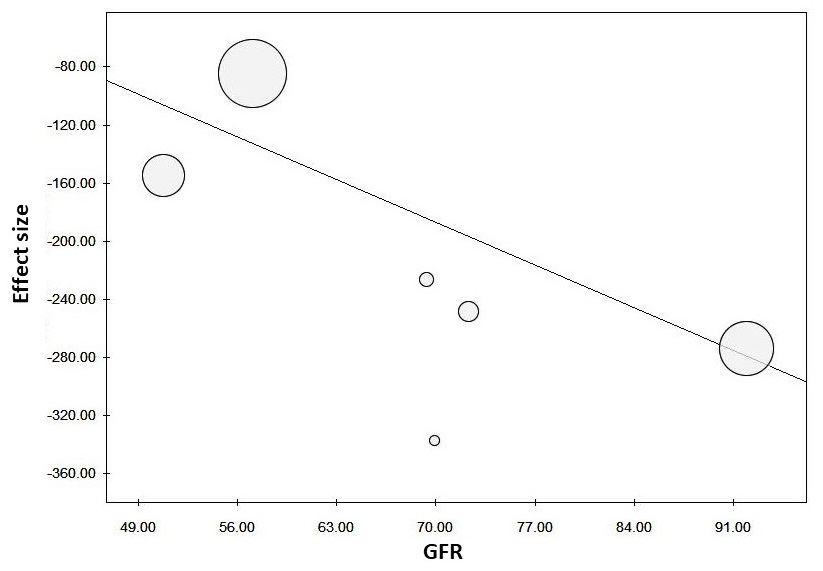


Supplemental Figure 2. Meta regression of differences in female gender percentage and overall unstandardized mean difference of six studies evaluating liver volume.
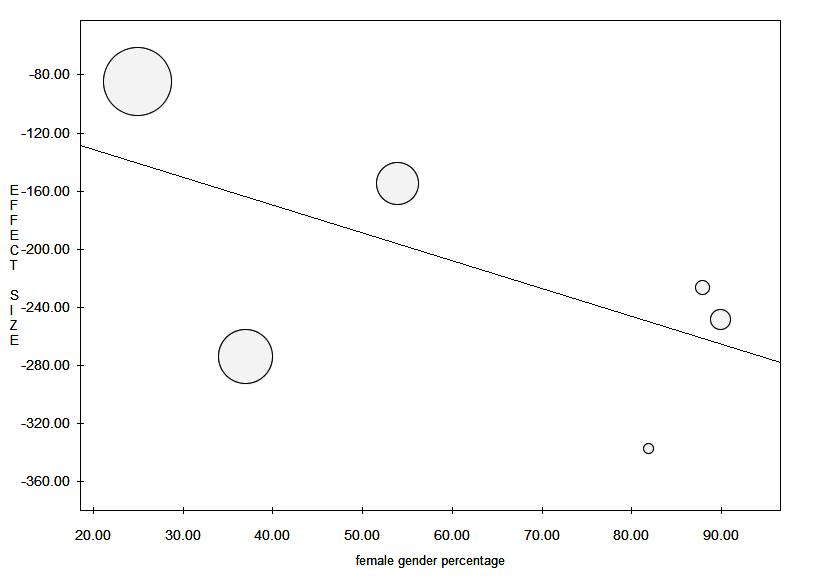


Supplemental Figure 3. Funnel plot for publication bias among studies evaluating the effects of somatostatin analogues on liver volume.


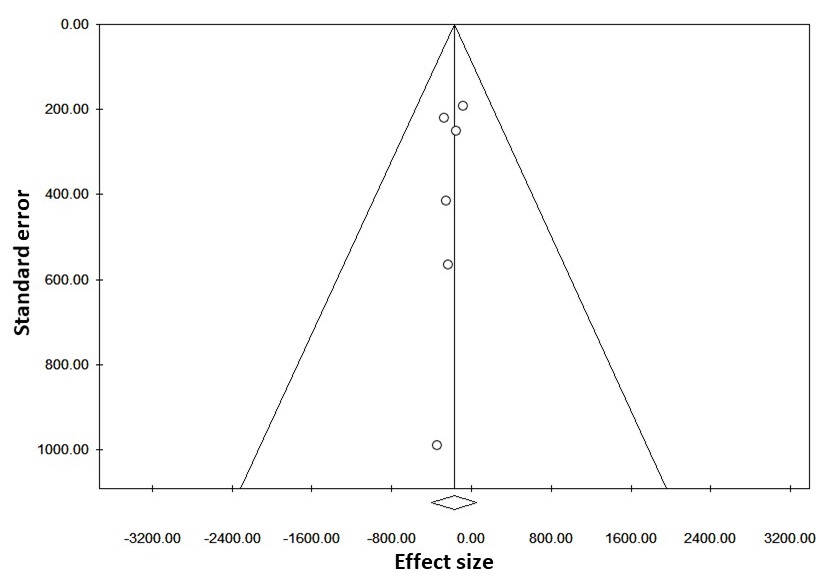


**Supplemental Table 1.** Baseline and final liver volume in somatostatin analogue group and placebo/standard management with values of unstandardized mean difference considering a pre-post correlation of 0.5, 0.7 and 0.9.

| **Authors** | **Liver volume baseline SA** | **Liver volume**  **Final SA** | **Liver volume baseline placebo/SM** | **Liver volume**  **Final placebo/SM** | **Pre-post correlation** | **UMD** | **P** | **Pre-post correlation** | **UMD** | **P** | **Pre-post correlation** | **UMD** | **P** |
| --- | --- | --- | --- | --- | --- | --- | --- | --- | --- | --- | --- | --- | --- |
| Van Keimpema | 4606±2071 | 4471±2005 | 4804±2032 | 4896±2121 | 0.5 | -227  [-1335, 881] | 0.688 | 0.7 | -227  [-1086, 632] | 0.604 | 0.9 | -227  [-724, 270] | 0.371 |
| Caroli | 1595±478 | 1524±453 | 1580±487 | 1594±480 | 0.5 | -85  [-465, 295] | 0.661 | 0.7 | -85  [-379, 209] | 0.572 | 0.9 | -85  [-255, 85] | 0.328 |
| Hogan (2010) | 5908±2915 | 5557±2659 | 5374±3565 | 5361±3331 | 0.5 | -338  [-2279, 1603] | 0.733 | 0.7 | -338  [-1845, 1169] | 0.660 | 0.9 | -338  [-1218, 542] | 0.451 |
| Pisani | 1610±501 | 1480±471 | 1693±471 | 1837±749 | 0.5 | -274  [-708, 159] | 0.214 | 0.7 | -274  [-623, 74] | 0.122 | 0.9 | -274  [-508, -41] | **0.021** |
| Van Aerts | 3434±1816 | 3376±1855 | 2811±1227 | 2908±1184 | 0.5 | -155  [-647, 337] | 0.537 | 0.7 | -155  [-537, 226] | 0.425 | 0.9 | -155  [-376, 65] | 0.168 |
| Hogan (2020) | 2582±1381 | 2479±1317 | 2387±759 | 2533±770 | 0.5 | -249  [-1066, 568] | 0.550 | 0.7 | -249  [-882, 384] | 0.441 | 0.9 | -249  [-616, 118] | 0.183 |

**SA, somatostatin analogues; SM, standard management; UMD, unstandardized mean difference.**
